# RIM1/2 in retinal ganglion cells are required for the refinement of ipsilateral axons and eye-specific segregation

**DOI:** 10.1038/s41598-017-03361-0

**Published:** 2017-06-12

**Authors:** Ahlem Assali, Corentin Le Magueresse, Mohamed Bennis, Xavier Nicol, Patricia Gaspar, Alexandra Rebsam

**Affiliations:** 1 Institut National de la Santé et de la Recherche Médicale, UMR-S 839, 75005 Paris, France; 2 Université Pierre et Marie Curie, 75005 Paris, France; 3 Institut du Fer à Moulin, 75005 Paris, France; 4 Cadi Ayyad University, Marrakesh, Morocco; 5 UMR-S 968, Institut de la Vision, Paris, France

## Abstract

Neural activity is crucial for the refinement of neuronal connections during development, but the contribution of synaptic release mechanisms is not known. In the mammalian retina, spontaneous neural activity controls the refinement of retinal projections to the dorsal lateral geniculate nucleus (dLGN) and the superior colliculus (SC) to form appropriate topographic and eye-specific maps. To evaluate the role of synaptic release, the rab-interacting molecules (RIMs), a family of active zone proteins that play a central role in calcium-triggered release, were conditionally ablated in a subset of retinal ganglion cells (RGCs). We found that this deletion is sufficient to reduce presynaptic release probability onto dLGN neurons. Furthermore, eye-specific segregation in the dLGN and topographic refinement of ipsilateral axons in the SC and the dLGN, are impaired in RIM1/2 conditional knock-out (Rim-cDKO) mice. These defects are similar to those found when retinal activity is globally disturbed. However, reduction in synaptic release had no effect on eye-specific lamination in the SC nor on the retinotopic refinement of contralateral axons in the SC. This study highlights a potential distinction between synaptic and non-synaptic roles of neuronal activity for different mapping rules operating in visual system development.

## Introduction

Refinement of specific neural connections during development requires axon branching at the appropriate location and the elimination of improper collaterals. The role of activity in these processes has been extensively studied in the visual system^1^, but most studies have focused on perturbing retinal activity^2–6^ without identifying the downstream mechanisms. In mammals, retinal ganglion cells (RGC) project to their main targets, the superior colliculus (SC) and the dorsal lateral geniculate nucleus (dLGN) as two organized maps: an eye-specific map with segregated ipsilateral and contralateral RGC projections and a retinotopic map with neighboring RGCs connecting neighboring target cells. Ipsilateral and contralateral projections overlap at birth and then segregate to form eye-specific domains during the first postnatal week^7, 8^. Achieving precise retinotopy involves the retraction of retinal projections that have initially overshot their termination zone, the branching at the proper location and the axonal arbor refinement to focus the termination zone^5, 6, 9^.

During the establishment of these retinal maps, waves of spontaneous and correlated neuronal activity among neighboring RGCs occur in the retina^10, 11^ and are transmitted to brain targets^12^ through synaptic release of glutamate^13^. Perturbing spontaneous retinal activity leads to defects in both eye-specific segregation and retinotopic refinement^2–6, 14, 15^. Neurotransmission at retinogeniculate and retinocollicular synapses is thought to drive the connectivity refinement through synaptic competition between axons^16–19^, however the involvement of synaptic release mechanisms has not been directly evaluated.

To specifically question the role of calcium-dependent synaptic release from RGCs for visual map establishment, we targeted the deletion of the rab-interacting molecules (RIMs), a family of proteins in the active zone of the synapse that are involved in vesicle docking, priming^20^ and in tethering calcium channels^21^. RIM1 protein is needed for presynaptic long-term potentiation (LTP)^22, 23^. Removal of all RIM 1 and 2 isoforms by the combined deletion of the *Rim1* and *Rim2* genes leads to a strong reduction of the calcium-dependent synaptic release ranging from 70 to 90% according to the synapses investigated^20, 21, 24–27^. Here, we show that the presynaptic release probability on dLGN neurons is indeed reduced after conditional deletion of RIM1/2 in a fraction of RGCs. This is sufficient to cause eye-specific segregation defects in the dLGN and abnormal refinement of ipsilateral projections in both the dLGN and the SC, similarly to models where retinal activity is disturbed. However, both eye-specific lamination and retinotopic refinement of contralateral projections from the ventro-nasal retina were normal in the SC in our model of conditional RIM1/2 deletion. All together, these results help identifying features of activity-dependent retinal map refinement that are differentially sensitive to synaptic release mechanisms.

## Material and Methods

### Animals

The Rim1^flox/flox^ Rim2^flox/flox^ mouse line^21, 28^ is a gift from P. Kaeser and T. Sudhof (Stanford university, USA). The serotonin transporter (Sert/Slc6a4) Cre mouse line is a knock-in of NLS-Cre in the 5′UTR region of Sert/Slc6a4 gene^29^. Sert^Cre/+^ Rim1^flox/−^ Rim2^flox/−^ males^25^ were crossed to Rim1^flox/flox^ Rim2^flox/flox^ females. Sert^Cre/+^ offsprings are referred to as Rim conditional double knock-out (Rim-cDKO) and Sert^+/+^ offsprings are referred to as controls (Ctrl), regardless to their Rim1 and Rim2 genotypes (flox/− or flox/flox). Genotyping was done as described in ref. 25. For the recombination study, the Sert Cre mouse line was crossed to the reporter mouse line Tau^mGFP-NLS-LacZ^ ^30^. All the experiments were approved (animal protocol #2496) by the French Ministry of Agriculture and Forestry, and conducted in compliance with the European community ethical guidelines - decree 2010/63/UE.

### Anterograde labeling of retinogeniculate and retinocollicular projections

Adult mice were anesthetized with ketamine-xylazine (93.75 mg/kg and 12.5 mg/kg respectively, in 0.9% saline). Pups were anesthetized on ice during 5–8 min. For whole-eye labeling, eyes of P6 and P24 mice were injected with a glass micropipette intravitreally with 2–3 μl of 0.2% cholera-toxin subunit B (CTB; Molecular Probes) conjugated to AlexaFluor 594 or 488 diluted in 1% DMSO. Focal injections of liquid DiI (Molecular probes, 0.4% in 4% dimethylformamide and 10% sucrose) into the ventro-nasal retinal quadrants of P13 mice were performed using a Picospritzer® III pressure injection system. 2 to 3 days after CTB or DiI injection respectively, mice were anesthetized with pentobarbital (545.7 mg/kg) and perfused transcardially with 4% paraformaldehyde (PFA) in 0.12 M phosphate buffer (PB). For CTB injections, brains were postfixed overnight in 4% PFA, cryoprotected 2 days in 30% sucrose in PB, sectioned with a freezing microtome (60 μm) and mounted in Mowiol DabCo (Calbiochem, Sigma). For DiI injections, brains and eyes were postfixed overnight in 4% PFA. Whole superior colliculi and retinas were dissected and mounted in Mowiol DabCo.

### Immunohistochemistry

Sert^Cre/+^ Tau^mGFP-NLS-LacZ^ P7 mice were euthanized with an overdose of pentobarbital-xylazine and perfused transcardially with 4% PFA in 0.12 M PB. Eyes were postfixed overnight in the same fixative, cryoprotected with 10% sucrose in PB during 2 days. Eyes were then embedded with 7.5% gelatin and 10% sucrose in PB, frozen 1 minute in isopentane at −55 °C, sectioned with a cryostat (20 µm; Leica CM 3000) and collected on superfrost slides. Slides were stored at −80 °C.

Sections were blocked with PGTx (0.2% gelatin and 0.25% Triton X-100 in PBS). Antibodies were diluted in PGTx: Rabbit anti-βGal (1/5000, Rockland), Goat anti-ChAT (1/200, Chemicon), Donkey anti-rabbit 488 and Donkey anti-goat Cy3 (1/500; Jackson Immunoresearch).

For retinal whole-mount immunostaining, retinas were first permeabilized in 1% Triton X-100 for 30 minutes, blocked in PHTx (0.1% Triton X-100 10% horse serum in PBS). Antibodies were diluted in PHTx: Rabbit anti-βGal (1/5000, Rockland), Goat anti-Brn3 (1/200, C-13, Santa Cruz), Rabbit anti-RBPMS (1/500, Phosphosolutions, # 1830), Donkey anti-rabbit AlexaFluor488, Donkey anti-chicken AlexaFluor488, Donkey anti-rabbit Cy3 and Donkey anti-goat Cy3 (1/200, Jackson). The anti-Brn3 antibody marks the Brn3a, b and c, leading to the labeling of 80% of RGCs^31^.

### Recombination analysis

Confocal images (TCS SP5 II, Leica) were acquired in each part (periphery, middle, center) of the 4 retinal quadrants of flattened retinas with a 40X objective. Using Cell Counter of Image J software, we counted the number of βGal + Brn3 + cells and Brn3 + cells in P7 Sert^Cre/+^ Tau^mGFP-NLS-LacZ^ mice (n = 4).

### Electrophysiology

dLGN slices were prepared from control and Rim-cDKO mice (P14-P16). Mice were killed by decapitation. The brain was removed and submerged in ice-cold ACSF containing (in mM): 125 NaCl, 25 NaHCO_3_, 1.25 NaH_2_PO_4_, 2.5 KCl, 2 CaCl_2_, 1 MgCl_2_, 25 glucose, continuously bubbled with 95% O_2_ and 5% CO_2_ (pH 7.3). Coronal slices (300 µm) were cut using a vibratome (HM650V, Thermo Scientific). Slices were stored at room temperature and transferred for patch-clamp recordings to a recording chamber where they were superfused with ACSF at 30–32 °C. Whole-cell voltage-clamp recordings were performed using pipettes pulled from borosilicate glass capillaries with a resistance of 4–6 MΩ when filled with the following solution (in mM): 120 Cs-gluconate, 10 CsCl, 8 NaCl, 10 HEPES, 10 phosphocreatine-Na, 2 MgATP, 0.2 EGTA (pH 7.3, adjusted with CsOH). The recorded neurons were clamped at a holding potential of −65 mV. The liquid junction potential (−15 mV) was not corrected. Membrane capacitance and series resistance were not compensated. GABA_A_ receptors were blocked with 10 μM SR95531 hydrobromide (gabazine, Hello Bio). Stimulus delivery was performed with a stimulation electrode located in the vicinity of the optic tract. Data acquisition was performed using an EPC10 amplifier and PatchMaster software (HEKA). Signals were sampled at 20 kHz, filtered at 5 kHz, and off-line analysis was performed using Igor Pro (WaveMetrics). The paired-pulse ratio is defined for each cell as the mean amplitude of the second EPSC divided by the mean amplitude of the first EPSC for 10–16 consecutive traces recorded at 0.1 Hz.

### Analysis of the distribution and segregation of ipsilateral fibers in the dLGN and SC

Quantifications were performed on images obtained with a 10X/0.40 objective of a Leica DM 6000B epifluorescence microscope from three consecutive coronal sections containing the greatest extent of the ipsilateral projection for the dLGN and on three consecutive coronal sections from either the rostral or the intermediate SC. We measured the overlap of ipsilateral and contralateral pixels in the dLGN after thresholding, the percentage of ipsilateral signal (number of ipsilateral pixels/number of pixels within the dLGN or the SC) and the extent of the ipsilateral projections along the mediolateral and dorsoventral axis in the dLGN using Metamorph software (Molecular Devices) as in Rebsam *et al*.^3^. Furthermore, we measured the “ipsilateral region” that represent the region of the dLGN encompassing all ipsilateral projections (dense and sparse) by tracing a manual contour around the ipsilateral projection and expressed as a percentage of the dLGN area. Statistical analyses were performed using an ANOVA test for the co-localization at different threshold and a Mann-Whitney test for the size, extension and area of ipsilateral axons.

### Analysis of the retinotopy of contralateral fibers

We examined all retinas after DiI injection to confirm the position of the injection in retinal quadrants, and to discard inappropriate injections when these were either too small (no labeling) or too large (more than one fourth of the retina labeled). Images of whole flattened dorsal SC were captured with a fluorescent macroscope (Olympus MVX10X). The size of the DiI patch was measured as a ratio of DiI pixels (after image threshold) to the total number of pixels in the SC, blind to genotype using Metamorph. Statistical analyses were performed using a Mann-Whitney test.

## Results

### Recombination occurs in more than 24% of RGCs at P7 in the Rim-cDKO model

**Figure 1:**
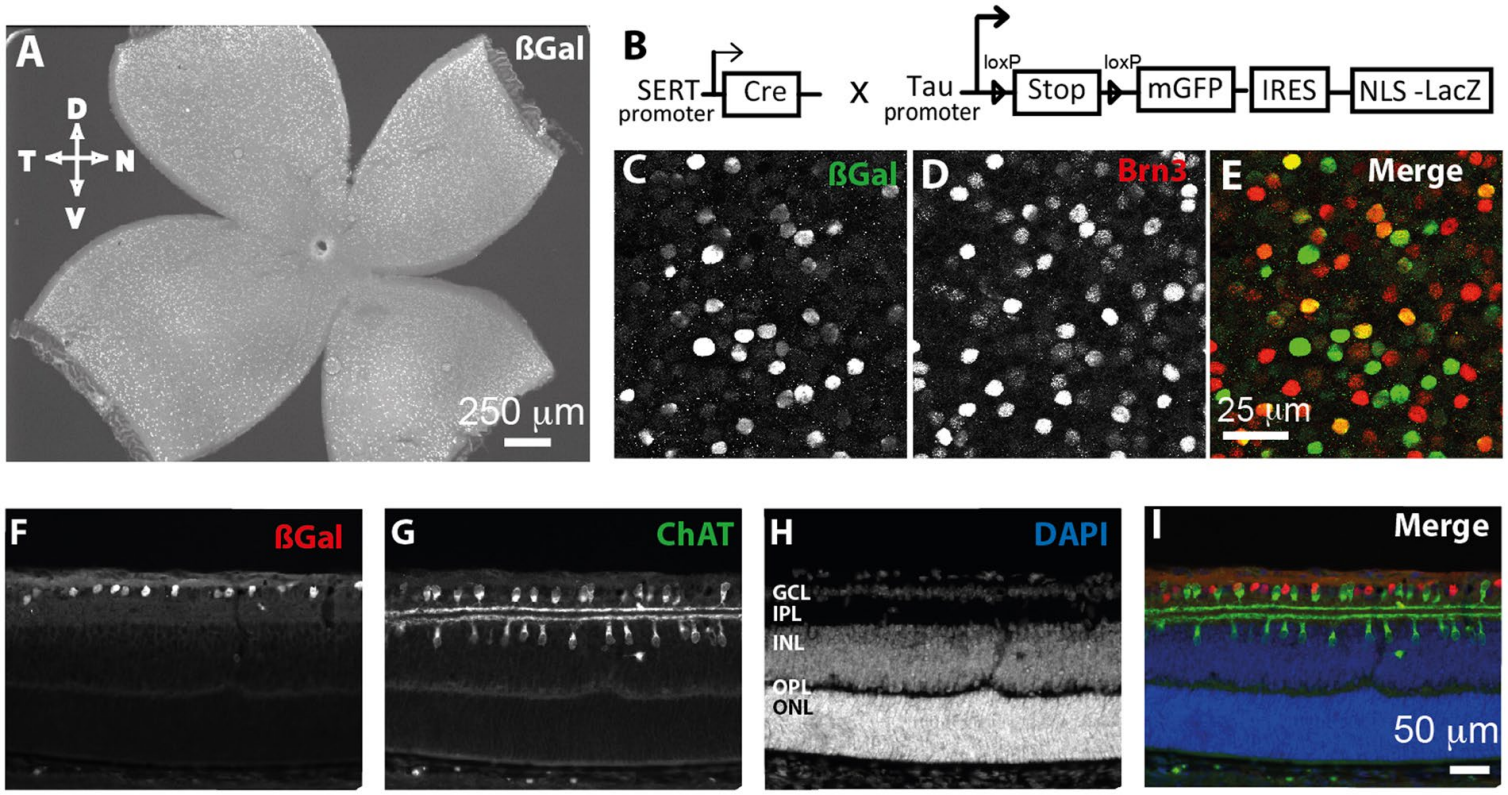
Cre recombination in the retina of P7 Sert-Cre mice. (**B**) Description of the reporter mouse line Sert ^Cre/+^ Tau^mGFP-NLS-LacZ/+^ used to identify the cells expressing Cre recombinase. (**A**) Whole-mount retina at P7 after βGal immunostaining and inset with βGal (**C**) and Brn3 (**D**) co-immunostaining (**E**). βGal (**F**), ChAT (**G**) and DAPI (**H**) stainings on retinal sections in Sert^Cre/+^ Tau^mGFP-NLS-LacZ/+^ mice at P7. Merged image (**I**) shows that most recombined retinal cells are located in the ganglion cell layer (GCL) and excluded from displaced starburst amacrine cells (ChAT+) located in this layer.

As the serotonin transporter gene (SERT, *Slc6a4*) has been previously found to be expressed in the retina by E15 and restricted to the peripheral ventro-temporal crescent of the retina in the RGCs projecting ipsilaterally^32, 33^, we used a mouse line in which Cre expression is driven by the serotonin transporter promoter (SERT Cre)^29^, with the initial goal to delete RIMs only in ipsilateral RGCs. Even though the recombination of reporter alleles may not be exactly the same as recombination of the floxed alleles RIM1/2, the Tau^mGFP-NLS-LacZ^ reporter mouse line (Fig. 1B)^30^ allows us to see where Cre recombinase is expressed in our SERT Cre driver line and to identify the cell bodies using βGal immunostaining and axons using GFP immunostaining. Based on Sert/Slc6a4 expression in the retina during early postnatal life^32, 33^, we expected expression to be only or mainly in ipsilateral RGCs, however, we observed that recombination occurred across the entire retina in Sert^Cre/+^ Tau^mGFP-NLS-LacZ^ (Fig. 1A). As refinement of the retinal projections occurs during the first postnatal weeks (P3-P14), we investigated the degree of recombination in the retina during development, at P7 using cell-type specific retinal markers. βGal-positive recombined cells were found only in the RGC layer (Fig. 1F,H,I) and exclusively in the RGCs as shown with the RGC-specific marker Brn3, which labels 80% of all RGCs^31^ (Fig. 1D). 30.4% ( ± SD:5.6%) of the Brn3-positive RGCs were βGal-positive. As 20% of RGCs are Brn3-negative, the total number of βGal-positive cells can be estimated to range between 24% (if none of the Brn3-negative RGCs were recombined) and 44% (if all Brn3-negative RGCs were recombined). These data thus suggest that at least 24% of RGCs have effectively recombined by P7 (Fig. 1C–E). Conversely, βGal staining was not present in the displaced starburst amacrine cells labeled with Choline Acetyl Transferase (ChAT) (Fig. 1F–I). Thus, ACh neurotransmission from starburst amacrine cells to RGCs, involved in spontaneous retinal activity, should not be affected in our model. Immunohistochemistry for βGal and RBPMS, a marker of all RGCs^34^ on whole-mount retina of Sert^Cre/+^ Tau^mGFP-NLS-LacZ^ mice at P10 showed that all βGal-positive cells are indeed RBPMS-positive (Suppl. Fig. 1D–F), confirming that the recombination occurs only in RGCs in the retina. Furthermore, RGC retrograde tracing using stereotaxic injection of fluorogold in the dLGN of adult mice showed that 98% of the fluorogold retrogradely labeled RGCs were βGal-positive, with a similar density in all retinal quadrants (Suppl. Fig. 1A–C). Thus, in the adult, nearly all RGCs projecting to the dLGN are likely recombined, suggesting that the recombination may occur more often in RGCs that project to the dLGN. Beside the retina, Cre recombination occurs also in other components of the visual system, as shown by βGal-positive cells in the dLGN and the primary visual cortex (V1), but not in the SC (Suppl. Fig. 2) consistent with previous reports^25, 29, 35^.

### Presynaptic release from retinal axons onto dLGN neurons is reduced in Rim-cDKO

**Figure 2:**
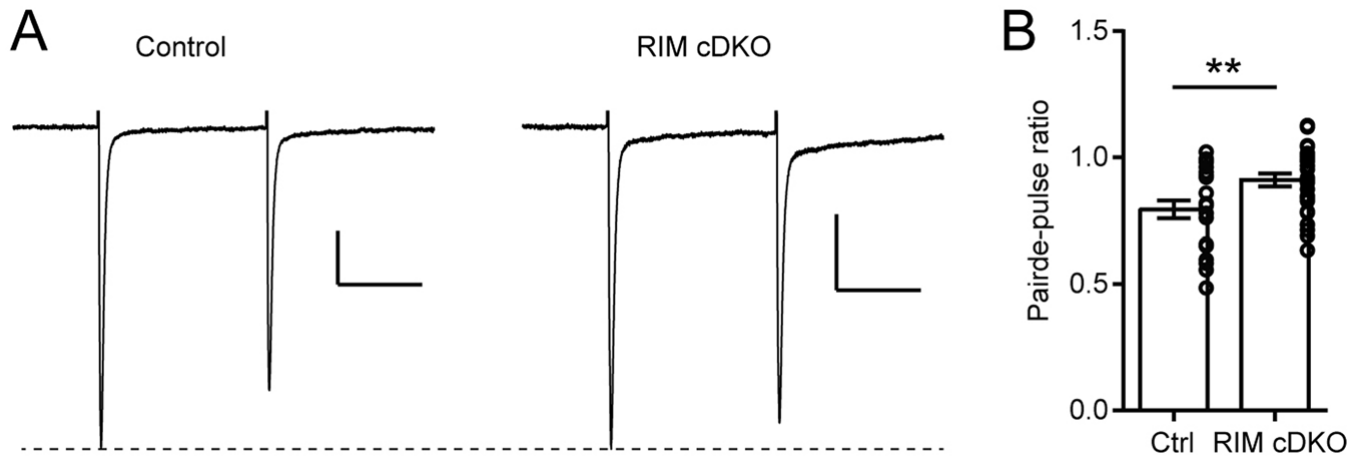
Decreased probability of glutamate release at the retinogeniculate synapse in Rim-cDKO mice. (**A**) Sample evoked excitatory postsynaptic currents recorded in dLGN neurons in response to paired stimulations of the optic tract (interstimulation interval 100 ms). Neurons were held at a holding potential of −65mV. Each trace is the average of 16 consecutive recordings at 0.1 Hz. The first EPSC of each trace was scaled for comparison. Scale bars: 50 pA and 50 ms. (**B**) Summary histogram showing the paired-pulse ratio (mean ± SEM; control 0.79 ± 0.04 versus Rim-cDKO 0.91 ± 0.03), defined as the mean amplitude of the second EPSC divided by the mean amplitude of the first EPSC for 10–16 consecutive traces recorded at 0.1 Hz. Each open circle represents the calculated PPR value for one individual cell. Control: n = 22 cells from 3 mice. Rim-cDKO: n = 28 cells from 4 mice. **p = 0.009, 2-tailed T-test

To determine whether RIM1/2 deletion in a subset of RGCs affects presynaptic release onto dLGN neurons, we performed whole-cell patch-clamp recordings of dLGN neuron in P14-P16 mice and measured the paired-pulse ratio (PPR) of postsynaptic currents evoked by stimulation of the optic tract containing the retinal axons. The PPR is a quantification of the postsynaptic response depression that is observed for the second of two stimulation pulses applied in close succession and is a function of glutamate release probability. Although only a fraction of RGCs are affected by RIM1/2 deletion at this stage of development, the PPR was significantly higher in Rim-cDKO than in control mice (p = 0.009, 2-tailed t-test; Fig. 2), indicating that the probability of glutamate release from RGCs onto dLGN neurons was decreased in Rim-cDKO mice.

### Rim-cDKO show eye-specific segregation defects in the dLGN but not in the SC

Spontaneous activity in the retina is required for eye-specific segregation in both the dLGN and the SC, as has been shown using various models with disturbed retinal activity^1, 16–19^.

**Figure 3:**
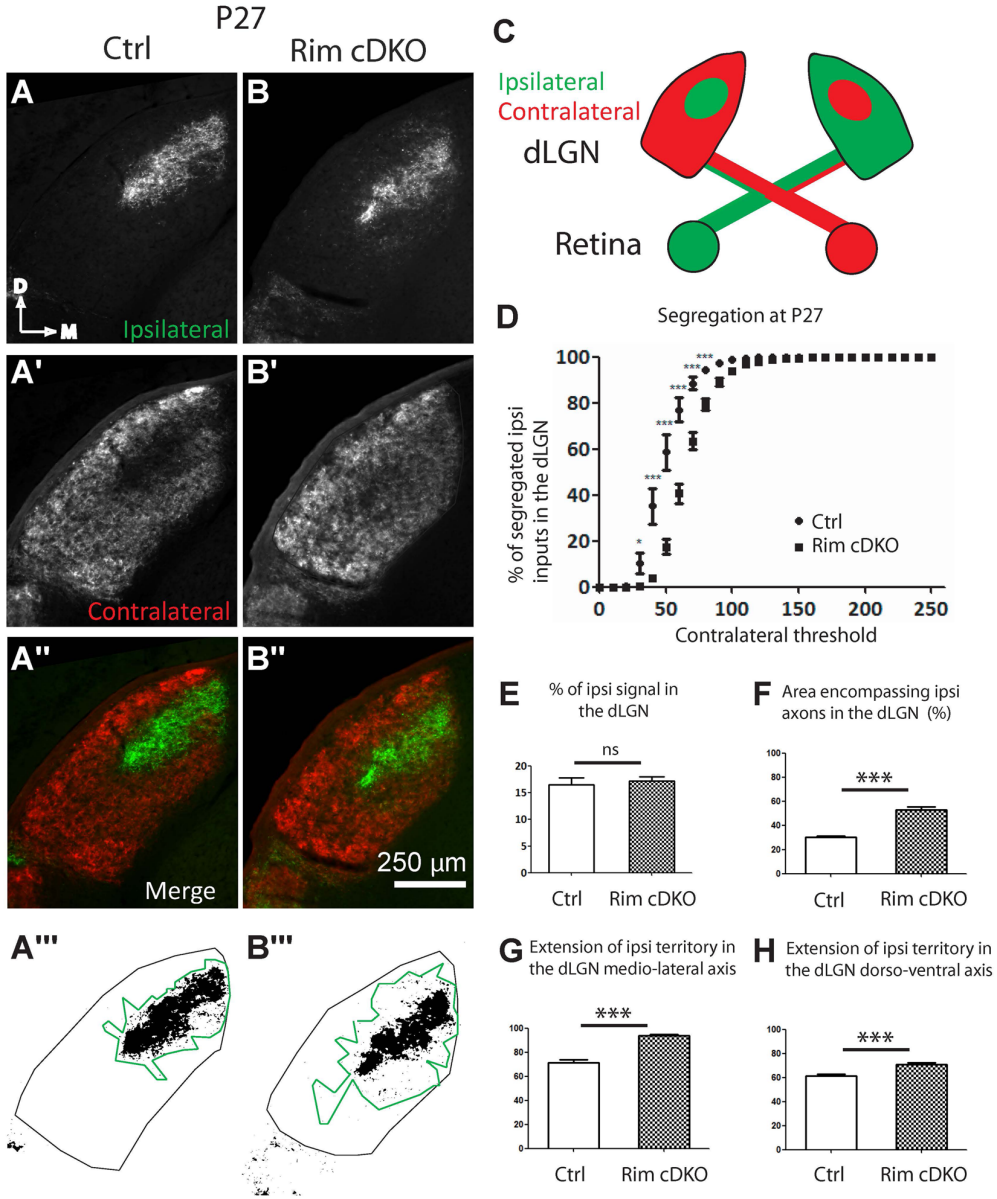
Eye-specific segregation in the dLGN at P27 requires RIM1/2. Anterogradely traced retinogeniculate projections (**A-B**″) in control (**A**-**A**″) and Rim-cDKO (**B**-**B**″) mice at P27. Ipsilateral (**A**,**B**) and contralateral (**A**′,**B**′) projections. Region encompassing ipsilateral axons within the dLGN on a binary image of ipsilateral signal in control (**A**″′) and Rim-cDKO (**B**″′) mice and quantified in F. (**C**) Schema representing the CTB-AlexaFluorDye injection in the eyes and the contralateral and ipsilateral projection in the dLGN in a coronal plane (**D**) Segregation plot: Percentage of segregated ipsilateral inputs as a function of contralateral threshold. Two-way ANOVA test revealed a significant eye-specific segregation defect in Rim-cDKO mice. *p < 0.05, ***p < 0.001. Errors bars: SEM. (**E**) Ratio of ipsilateral pixels to the total number of pixels in the dLGN. Mann-Whitney test revealed no difference between control and Rim-cDKO in terms of proportion of ipsilateral projections within the dLGN. (**F**) Quantification of the region encompassing all ipsilateral axons within the dLGN. Mann-Whitney test revealed that the ipsilateral territory is more extended in Rim-cDKO mice than in control mice. Error bars: SEM values. ***p = 0.0003. Extension of the ipsilateral territory along the medio-lateral axis (**G**) and along the dorso-ventral axis (**H**) of the dLGN. Mann-Whitney test revealed that the ipsilateral territory is more extended in both axis. Errors bars: SEM values. ***p = 0.0003. ns: non significant.

**Figure 4:**
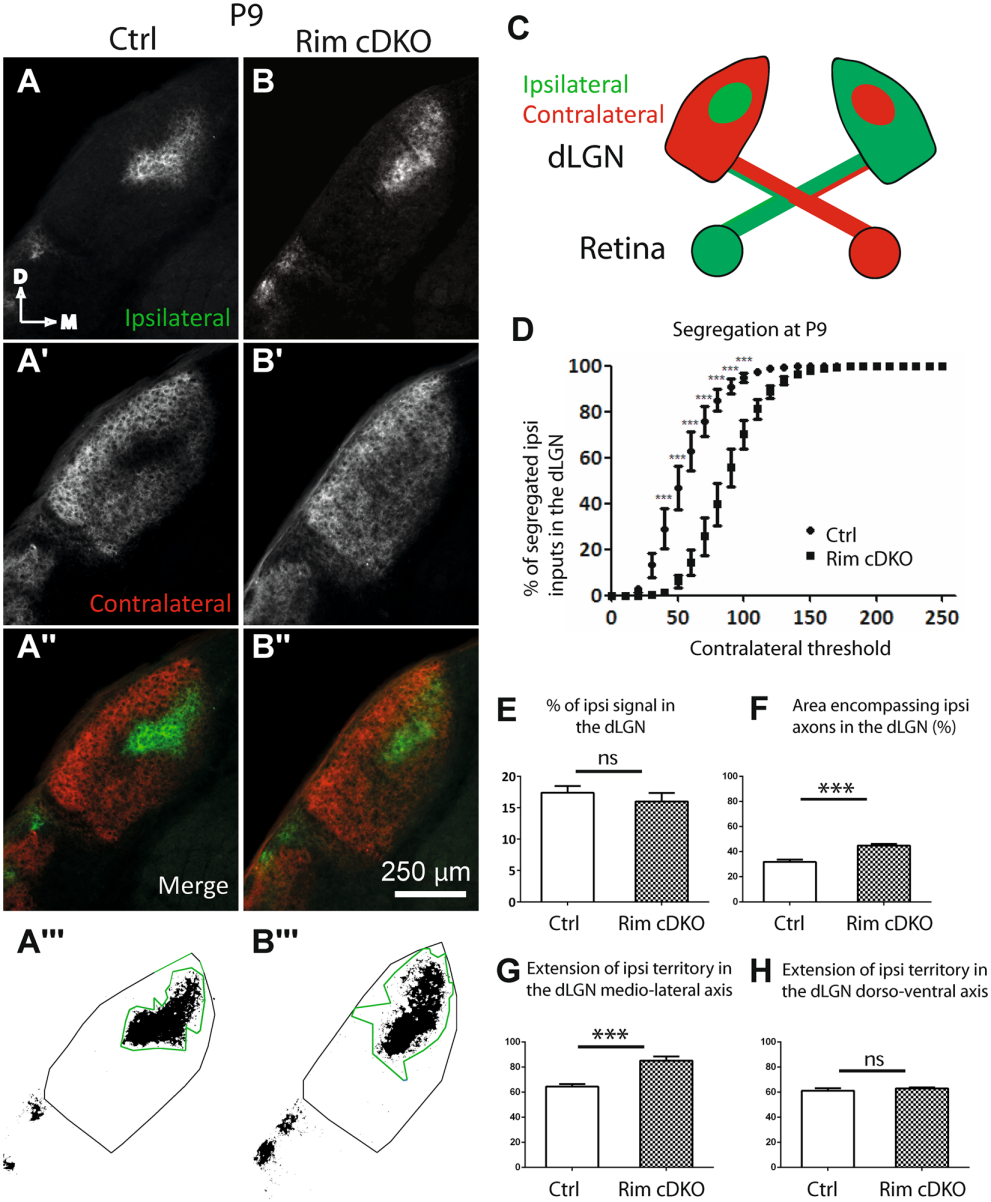
Eye-specific segregation in the dLGN at P9 requires RIM1/2. Anterogradely traced retinogeniculate projections (**A**-**B**″) in control (**A**-**A**″) and Rim-cDKO (**B**-**B**″) mice at P9. Ipsilateral (**A**,**B**) and contralateral (**A**′,**B**′) projections. Region encompassing ipsilateral axons within the dLGN on a binary image of ipsilateral signal in control (**A**″′) and Rim-cDKO (**B**″′) mice and quantified in F. (**C**) Schema representing the CTB-AlexaFluorDye injection in the eyes and the contralateral and ipsilateral projection in the dLGN in a coronal plane (**D**) Segregation plot: Percentage of ipsilateral segregated inputs as a function of contralateral threshold. Two-way ANOVA test revealed a significant eye-specific segregation defect in Rim-cDKO mice. ***p < 0.001. Errors bars: SEM. (**E**) Ratio of ipsilateral pixels to the total number of pixels in the dLGN. Mann-Whitney test revealed no difference between control and Rim-cDKO in terms of proportion of ipsilateral projections within the dLGN. (**F**) Quantification of the region encompassing all ipsilateral axons within the dLGN. Mann-Whitney test revealed that the ipsilateral territory is more extended in Rim-cDKO mice than in control mice. Error bars: SEM values. ***p = 0.0008. Extension of the ipsilateral territory along the medio-lateral axis (**G**) and along the dorso-ventral axis (**H**) of the dLGN. Mann-Whitney test revealed that the ipsilateral territory is more extended in the medio-lateral but not in the dorso-ventral axis. Errors bars: SEM values. ***p = 0.0008. ns: non significant.

To investigate the role of calcium-dependent synaptic release from RGCs in the formation of eye-specific domains, whole-eye anterograde labeling (CTB injections) was performed in control and Rim-cDKO mice (Figs 3 and 4). In P27 control mice, there was a gap in the contralateral projection (Fig. 3A′) corresponding to the ipsilateral projection (Fig. 3A). In Rim-cDKO, the contralateral projection covered the entire dLGN (Fig. 3B′), thus the ipsilateral and contralateral projections overlapped (Fig. 3B″) as confirmed by the reduction of the segregation of ipsilateral/contralateral terminals (Fig. 3D) at P27. This result suggests that contralateral axons are maintained in the ipsilateral territory. Similar defects were already observed at P9 (Fig. 4A,B) suggesting a developmental defect rather than a later remodeling defect of retinogeniculate axonal arborization. We demonstrated here that deletion of RIM1/2 in 24 to 44% of RGCs is sufficient to phenocopy the eye-specific segregation defects in the dLGN that are observed in the nicotinic beta2-receptor KO mice (beta2-KO)^2, 36^, a classical model with perturbation of spontaneous retinal waves of activity. This result suggests that in the dLGN, neural activity drives eye-specific segregation through synaptic release mechanisms and that interestingly, perturbing synaptic release only in a fraction of RGCs is sufficient to give rise to defects equivalent to those observed when the entire retinal activity is disturbed^2–4, 6^.

**Figure 5:**
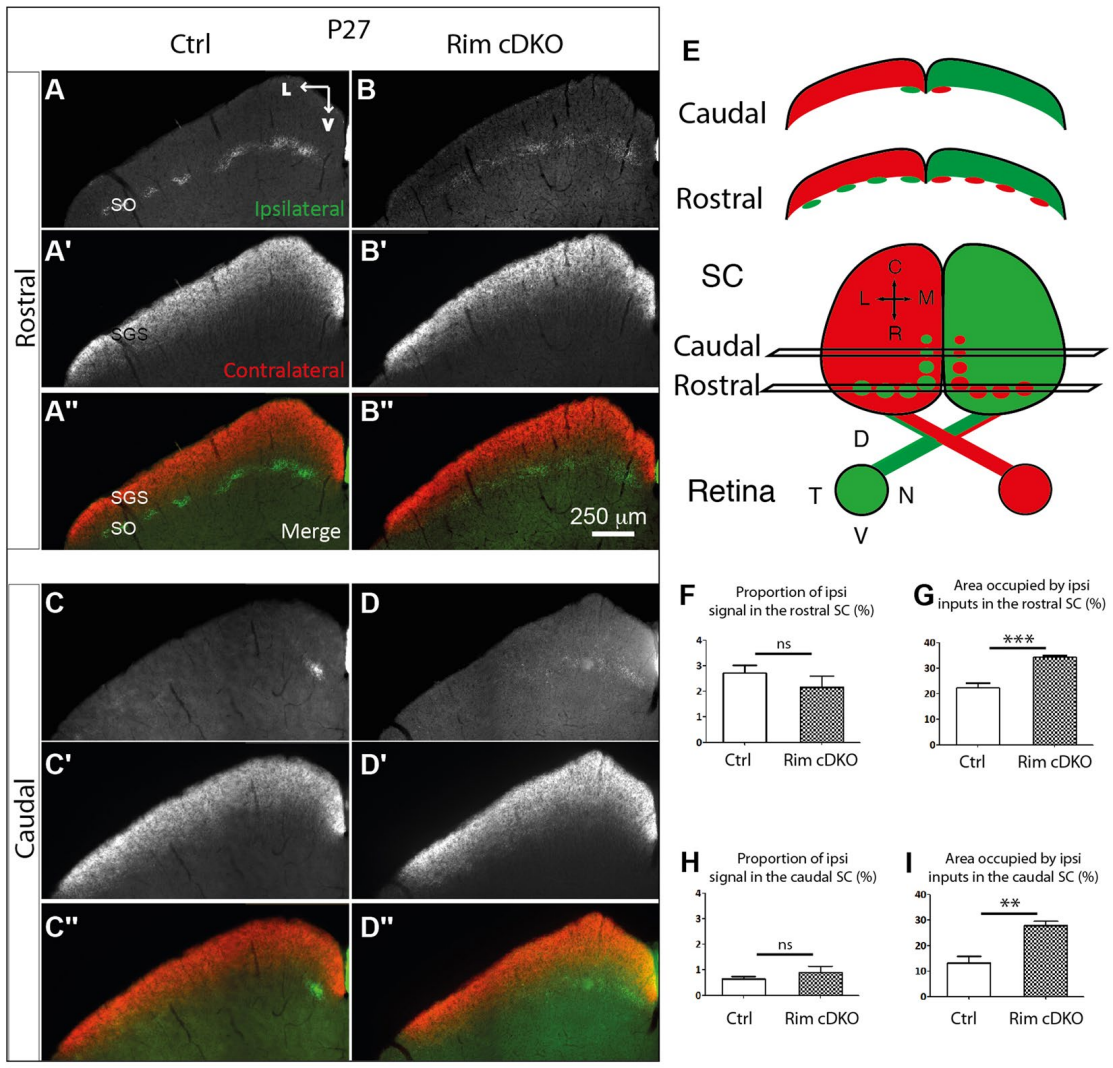
Ipsilateral retinocollicular projection requires RIM/2 for their topographic refinement. Anterogradely traced retinocollicular projections in control (**A**-**A**″, **C**-**C**″) and in Rim-cDKO (**B**-**B**″, **D**-**D**″) mice at P27. Ipsilateral (**A**,**B**,**C**,**D**) and contralateral (**A**′,**B**′,**C**′,**D**′) projections. (**E**) Schema representing the CTB-AlexaFluorDye injection in the eyes and the contralateral and ipsilateral projection in the SC at different rostro-caudal level. Ratio of ipsilateral pixels to the total number of pixels in the rostral (**F**) and caudal (**H**) SC. Mann-Whitney test revealed no difference in the proportion of ipsilateral projections between control and Rim-cDKO mice both in the rostral and caudal SC. Area occupied by the ipsilateral projection within the rostral (**G**) and caudal SC (**I**). Mann-Whitney test revealed that the ipsilateral territory is more extended in Rim-cDKO mice than in control mice both in the rostral and caudal SC. Error bars: SEM values. **p = 0.0012, ***p = 0.0003. ns: non significant.

We next examined whether similar segregation defects occur in the other major RGC target, the SC. In the SC, contralateral RGC axons project to the superficial part, the stratum griseum superficiale (SGS) (Fig. 5A′), whereas ipsilateral projections are restricted to a deeper layer, the stratum opticum (SO) (Fig. 5A). In Rim-cDKO mice, this eye-specific lamination occurred properly, each projection occupying the correct layer (Fig. 5B,B″). Thus in the SC, conditional removal of RIM1/2 in a fraction of RGCs does not phenocopy the eye-specific lamination defects found in the beta2-KO model^36^.

### The organization of ipsilateral projections in the dLGN and the SC is impaired in Rim-cDKO

Since retinal activity is important for the refinement of the ipsilateral projection in the dLGN and the SC^2, 36^, we next investigated the organization of the ipsilateral projection in Rim-cDKO. No significant difference was found in the proportion of ipsilateral axons between the Rim-cDKO and control mice at P9 and at P27, quantified in terms of ipsilateral pixel numbers over the total dLGN pixel numbers (Figs 4E and 3E), suggesting that the total number of ipsilateral terminals is not modified at this macroscopic level. However, we could observe that ipsilateral axons were spread over a larger dLGN region with scattered ipsilateral fibers extending away from the main ipsilateral region in Rim- cDKO compared to control. We found a significant expansion of the ipsilateral projection in the dLGN in P9 and P27 Rim-cDKO (n = 6 and n = 7, respectively) compared to control mice (n = 9 and n = 8, respectively) (Figs 3A,B and 4A,B), quantified in terms of the region encompassing all ipsilateral axons (in Fig. 3E and 4E) and in terms of the extent of ipsilateral axons along the medio-lateral axis (in Figs 3G and 4G). There was also a significant increase in the extent of ipsilateral axons along the dorso-ventral dLGN axis in P27 Rim-cDKO (Fig. 3H), but not in P9 Rim-cDKO (Fig. 4H). Overall, these quantifications confirm that the number of ipsilateral axons is normal but that they spread and are scattered over a larger area in the Rim-cDKO mice with ectopic ipsilateral terminations in the contralateral territory (Figs 3B,4B).

In the SC, at P3, ipsilateral retinal projections overshoot their termination zone, and then retract progressively up to P8-P10, to occupy only the very rostral portion of the SC, forming several patches along all the medio-lateral axis (Fig. 5A), and a single medial patch in the caudal SC (Fig. 5C)^7, 37^. In Rim-cDKO, the proportion of ipsilateral terminals was unchanged (Fig. 5F,H) but instead of being organized as patches (Fig. 5A,C), the projection was diffuse (Fig. 5B,D). The area occupied by ipsilateral inputs in the rostral SC of Rim-cDKO was increased compared to controls (Fig. 5G). Moreover, instead of being restricted to the medial part of the SC in more caudal sections of the SC (Fig. 5C), there was a significant lateral extension of the ipsilateral projection in Rim-cDKO (Fig. 5D) compared to the control area occupied by ipsilateral inputs (Fig. 5I).

Taken together, these results suggest that RIM1/2 are necessary to establish the organization of ipsilateral axons in the dLGN and in the SC. Moreover, these ipsilateral projection defects in the Rim-cDKO are similar to the defects found in the beta2-KO^2, 36^ suggesting that perturbing the presynaptic release via deletion of RIM1/2 in only a fraction of RGCs is sufficient to trigger a phenotype similar to global activity perturbed models (reviewed in Assali *et al*.)^1^.

### Contralateral projections in the medio-caudal SC exhibit unaffected retinotopy and refinement of termination zone in Rim-cDKO

**Figure 6:**
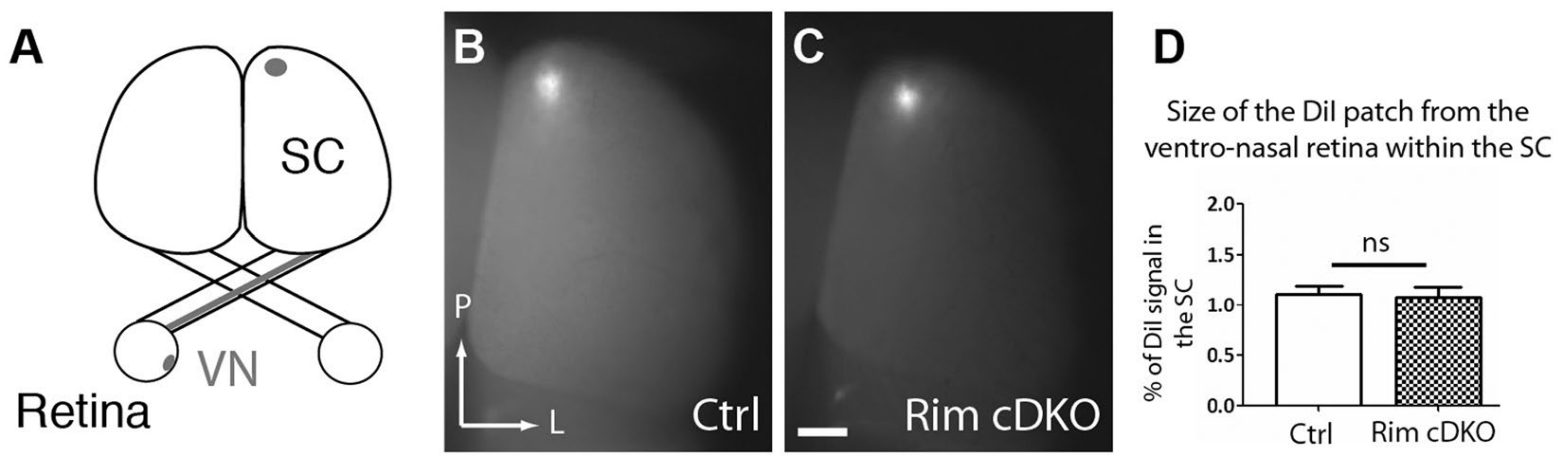
Normal retinotopic refinement of contralateral projections in the medio-caudal SC. (**A**) Schema representing the focal DiI injection in ventro-nasal (VN) retina and the retinotopic location of the retinocollicular projection in whole-mount view **(B**,**C**) Dorsal view of whole SC at P15. Focal target spot in the medio-caudal part of the contralateral SC after a focal DiI injection into VN retina in control (**B**) and in Rim-cDKO (**C**). Mann Whitney test revealed no difference in the size of the DiI patch in Rim-cDKO compared to control mice. Error bars: SEM values. ns: non significant. Scale Bar: 500 μm

After the axon guidance phase, spontaneous retinal activity is required to focus the termination zone formed by neighboring RGC projections^5, 6^. To determine whether topographic mapping of contralateral projections occurs normally when presynaptic release is perturbed, we performed focal DiI injections in the ventro-nasal part of the retina of control (n = 9) and Rim-cDKO (n = 7) mice at P15 to label a small subset of retinotopic projections (Fig. 6A). In Rim-cDKO mice, the patch was correctly located in the SC with ventro-nasal RGCs projecting to the medio-caudal part (Fig. 6B,C). Furthermore, the size of the termination zone in Rim-cDKO was similar to control mice (Fig. 6D), suggesting that reduction in presynaptic release of a fraction of RGCs does not affect the topographic location and the refinement of ventro-nasal contralateral axons at this macroscopic level.

## Discussion

**Figure 7:**
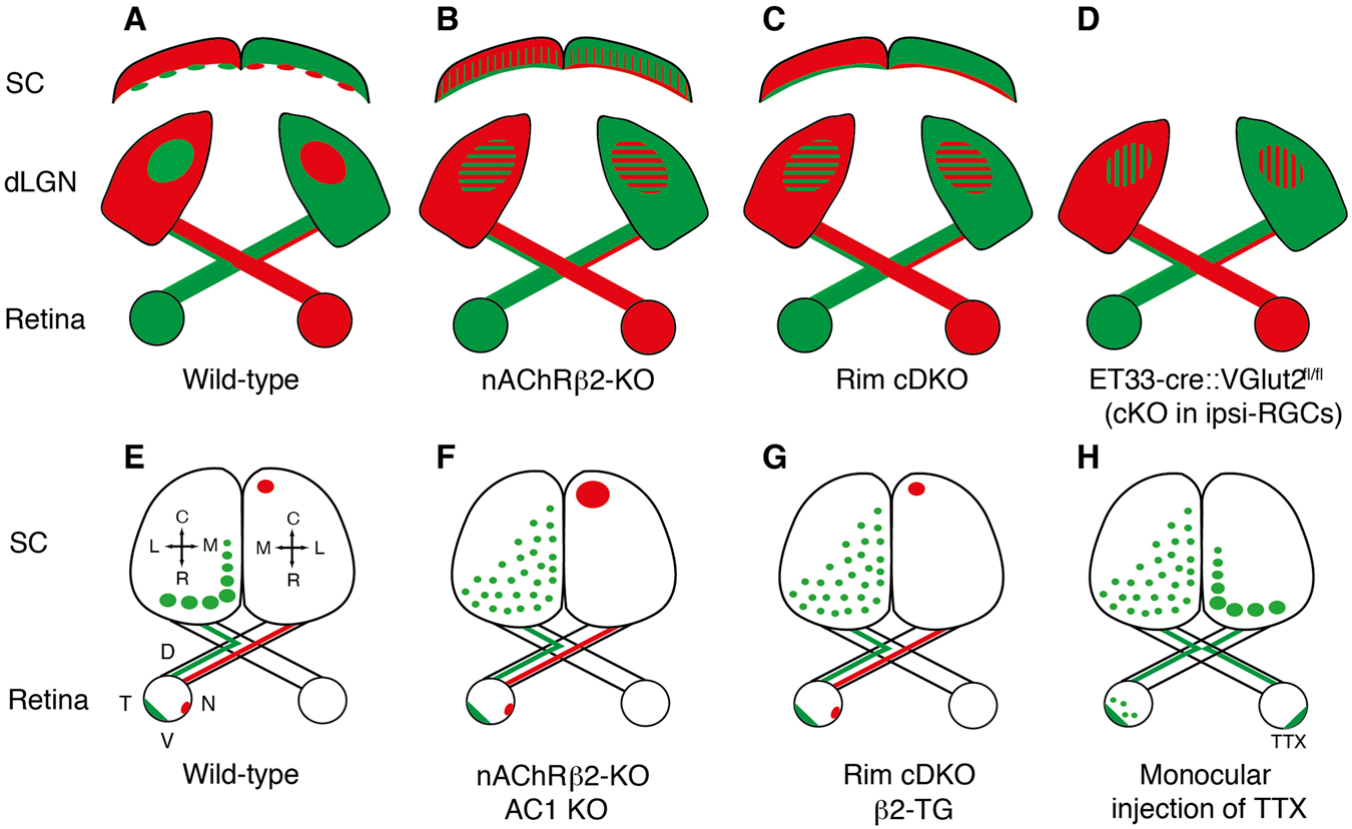
Effects of activity perturbation on eye-specific and topographic maps. (**A**–**D**) Schematic representations of whole retinal projections coming from the left eye (green) and the right eye (red) in the dLGN and in the rostral SC. Hatched regions correspond to regions where ipsilateral and contralateral axons overlap. (**E**–**H**) Topographic organization of ipsilateral (left) and contralateral (right) projections on a dorsal view of the SC. The entire ipsilateral projection was represented in green while the contralateral projections corresponding to focal injections into the ventro-nasal retina are represented in red. (**H**) Topographic organization of ipsilateral projections from both eyes after monocular intraocular injection of TTX. cKO: conditional knockout; KO: knockout; DKO: double knockout; dLGN: dorsal lateral geniculate nucleus; nAChR β2: beta2 subunit of the nicotinic acetylcholine receptor; RGCs: retinal ganglion cells; SC: superior colliculus; VGLUT2: vesicular glutamate transporter 2; TTX: Tetrodotoxin; C: caudal; L: lateral; R: rostral; M: medial; D: dorsal; T: temporal; V: ventral; N: nasal.

The present study indicated that RIM1/2 in a subset of RGCs are required for axonal branch elimination in the retinogeniculate and retinocollicular projections (Fig. 7C, G). While we cannot rule out that the exuberant projections correspond only to the RIM1/2-deleted RGCs, the similarity of the refinement defects observed in Rim-cDKO and in activity-perturbed models of the epibatidine-treated mice or the beta-2 KO mice (Fig. 7B)^2, 3, 38^ suggests a more global effect on retinogeniculate projections. Furthermore, we found that the presynaptic release probability of retinal axons onto dLGN neurons is reduced in Rim-cDKO mice, suggesting that retinal activity acts through synaptic release-dependent mechanisms for eye-specific segregation in the dLGN and the refinement of ipsilateral axons in retinocollicular and retinogeniculate projections. A previous study using perturbation of vesicular glutamate storage (VGLUT2-cKO) with a Cre-driver selective to the ipsilateral RGCs showed that glutamate-deficient ipsilateral axons failed to out-compete contralateral axons from the ipsilateral territory but were maintained (Fig. 7D)^39^. Thus, glutamate release by ipsilateral axons seems necessary for the proper retraction of contralateral axons. This is confirmed in the present study, where RIM1/2 are deleted in both ipsilateral and contralateral RGCs and the ipsilateral projection fails to effectively remove ectopic axonal projections from the contralateral territory. In addition the present model shows that presynaptic release from contralateral projections is required to refine the ipsilateral projection. Other observations indicate that neuronal activity in contralateral axons is necessary for the proper elimination of ipsilateral axons from caudal SC since monocular activity blockade of contralateral projections by tetrodotoxin (TTX), a blocker of sodium channels, modified the distribution of the non-treated ipsilateral projections in the SC (Fig. 7H)^40^, similarly to what is observed in Rim-cDKO mice (Fig. 7G). Thus, our present results and previous data strongly suggest a non-cell autonomous effect of synaptic transmission in both directions, with contralateral axons affecting ipsilateral axons and vice-versa. Further experiments where synaptic release is perturbed only in contralateral RGCs would be required to verify these hypotheses.

A potential limitation of our current genetic approach is that the SERT-cre driver induces recombination not only in RGCs but also in the relay cells of the dLGN that receives retinal inputs and project to the visual cortex, and in a fraction of neurons of the layer VI in the primary visual cortex (V1) that sends corticogeniculate axons to the dLGN. However, changes are unlikely at the level of the soma and dendrites of the relay neurons in the dLGN because RIM1/2 are localized at the active zone, presynaptically acting on calcium-dependent vesicular fusion^27, 41^ and indeed controlling presynaptic release of RGCs as we have shown here. Furthermore, we observe changes in the ipsilateral projections in Rim-cDKO in the SC, where Cre is not expressed (Suppl. Fig. 2C,D), strongly arguing for a role of RIM1/2 in the RGCs rather than in the target neurons. A recent report highlighted that corticogeniculate axons are required for the innervation of retinal axons into the dLGN^42^, raising the question of a potential role of RIM1/2 deletion in layer VI of the primary visual cortex in our model. However, this is very unlikely since the defects observed in the absence of layer VI neurons are completely different from the ones observed in our Rim-cDKO model. Indeed, when layer VI neurons are ablated, retinal axons fail to enter the dLGN^42^, while in Rim-cDKO retinal axons innervate properly and timely their target but then fail to eliminate exuberant axon branches. In fact, the perturbation of eye-specific segregation observed in Rim-cDKO is rather similar to the defaults observed in models with pharmacological perturbation of retinal activity or in beta2-cKO in the retina (Fig. 7B)^2, 3, 43, 44^, suggesting that perturbing activity only in RGCs can affect axon terminals as in our Rim-cDKO mice.

We showed that the presynaptic release probability from RGCs onto dLGN neurons is altered in Rim-cDKO, confirming previous data in other systems^20, 21, 24–27^ and highlighting the role of RIM1/2 on the regulation of synaptic release. Furthermore, we showed that RIM1/2 are important for the refinement of both contralateral and ipsilateral axons in their targets. This opens new questions to determine what are the mechanisms related to synaptic release that are crucial for these refinement events. One possibility is that the precise amount of glutamate released by retinal projections is important. Another non-exclusive possibility is that retinal projections release other molecules in addition to glutamate that are necessary for the refinement of retinal projections. A third possibility is that LTP-dependent mechanism could be required as RIM1 is required for presynaptic LTP^22, 23^. This latter possibility would also fit in well with the notion that correlation of synaptic release could be a key factor. Indeed, correlated activity in neighboring RGCs has an instructive role in eye-specific segregation in the dLGN^36^ and deletion of both RIM1/2 severely impairs the calcium responsiveness and synchronization of release^21, 24^. Thus, synaptic release is most likely uncorrelated to the features of spontaneous activity in these RIM1/2-deleted RGCs and this could be sufficient to globally affect axon terminal refinement.

Our study detected no change in the gross topographic position of contralateral retinocollicular axons in Rim-cDKO (Fig. 7E,G), suggesting that the topographic mapping of retinal axons in response to ephrin/Eph signaling is not dependent on presynaptic release, as expected from previous *in vitro* ^45^ and *in vivo* studies^6, 14, 15^. However, in activity-perturbed models like the beta2-KO, the termination zones of retinal axons were much wider than in controls^14^, suggesting that the refinement of retinal axons to a small and defined termination zone requires spontaneous retinal activity (Fig. 7E,F). Several models with conditional deletion of beta2 in the retina pinpoint to a role for local correlation of activity in neighboring RGCs for retinotopic refinement^36, 43, 44^. While we cannot rule out that at higher resolution, small defects could be visible in Rim-cDKO, present analyses show that the termination zone of contralateral axons from the ventro-nasal retina in the Rim-cDKO mice (Fig. 7G) was unchanged (Fig. 7E,F). Several interpretations can be offered. The first possibility is that, since our Cre driver abolished RIMs in only a fraction of the RGCs, even if synaptic release is necessary, this may not be sufficient to perturb topographic refinement in the caudal SC. The second possibility is that the general elimination of ectopic branches along the axons and the restriction of terminal arborization to a specific area requires retinal activity but does not require calcium-dependent synaptic release mechanisms. This interpretation is in agreement with observations on thalamocortical axonal arbors in the barrel cortex of Rim-cDKO^25^ that showed a normal organization. One potential mechanism that could drive the refinement of the termination zone independently of synaptic release is through AC1/cAMP signaling at the presynaptic level (Fig. 7F)^46–48^.

## Electronic supplementary material

Supplementary Information
