# Supplementary material for: RIM1/2 in retinal ganglion cells are required for the refinement of ipsilateral axons and eye-specific segregation
Source: Sci Rep. 2017 Jun 12;7:3236. doi: 10.1038/s41598-017-03361-0 (PMC5468276; doi:10.1038/s41598-017-03361-0)
Supplement: Supplementary file 1 — Supplementary Information [file 41598_2017_3361_MOESM1_ESM.pdf]

RIM1/2 are required in retinal ganglion cells for the refinement of ipsilateral axons and eye-specific segregation

Ahlem Assali [1,2,3], Corentin Le Magueresse [1,2,3], Mohamed Bennis [4], Xavier Nicol [1,2,3,5], Patricia Gaspar [1,2,3], Alexandra Rebsam [1,2,3, *]
[1]Institut National de la Santé et de la Recherche Médicale, UMR-S 839, 75005, Paris, France,
[2]Université Pierre et Marie Curie, 75005, Paris, France,
[3]Institut du Fer à Moulin, 75005, Paris, France,
[4]Cadi Ayyad University, Marrakesh, Morocco;
[5]Current address: UMR-S 968, Institut de la Vision, Paris, France

*Corresponding author: Alexandra Rebsam, INSERM U839, Institut du Fer à Moulin, 17 rue du fer à moulin, 75005, Paris, France. Phone: +33 1 45 87 61 27; Fax: +33 1 45 87 61 32; email: alexandra.rebsam@inserm.fr

Supplementary Material and Methods:

Retrograde tracing of retinal ganglion cells (RGCs)

$Sert^{Cre/+}$ $Tau^{mGFP-NLS-LacZ/+}$ adult mice (2 months) (n=x) were anesthetized with ketamine-xylazine (93.75mg/kg and 12.5 mg/kg respectively, in 0.9% saline) and fixed in a stereotaxic apparatus (Kopf). Glass micropipettes were pulled (tips broken) and filled with Fluorogold 5% (Molecular Probes) in 0.9% saline. Stereotaxically guided injections were performed in the right dorsal lateral geniculate nucleus (dLGN) through a hole in the dorsal surface of the cranium. Fluorogold injections (0.2-0.3µL) were targeted to the dLGN using the following coordinates : 2.5 mm caudal to the bregma, 2.2 mm lateral to the midline, and 2.5 mm depth from the brain surface. After injection with a nanoinjector (Narishige), micropipettes were left in place 10 min before removal to minimize leakage. After a 5-day survival, mice were perfused.

Immunohistochemistry

$Sert^{Cre/+}$ $Tau^{mGFP-NLS-LacZ}$ P4 (n=4), P10 (n=1) or adult mice (n=2) were euthanized with an overdose of pentobarbital-xylazine and perfused transcardially with 4% PFA in 0.12M PB. Brains and heads were postfixed overnight in PFA.

After retrograde labeling, brains were sectioned with a vibratome (100µm) and mounted in Mowiol DabCo to check the injection site. For beta-Gal and GFP immunhistochemistry, brains were cryoprotected 2 days in 30% sucrose in PB, sectioned with a freezing microtome (60µm) in coronal sections and mounted in Mowiol DabCo (Calbiochem, Sigma). Sections

were blocked with PGTx (0.2% gelatin and 0.25% Triton X-100 in PBS). Primary antibodies were diluted in PGTx: Rabbit anti-βGal (1/1000, Rockland), chicken anti-GFP (1/1000, Aves), Goat anti-FoxP2 (1/1000, SantaCruz).
Whole retinas (P10, adult) were oriented by an incision in the peripheral ventral part, dissected out from the head and rinsed in PBS. After retrograde labeling, retinas were blocked with PGTx (0.2% gelatin and 0.25% Triton X-100 in PBS) during an hour. Antibodies were diluted in PGTx: Rabbit anti-βGal (1/5000, Rockland), anti-Fluorogold (1:5000).
For RBPMS immunostaining, retinas were first permeabilized in 1% Triton X-100 for 30 minutes, blocked in PHTx (0.1% Triton X-100 10% horse serum in PBS). Antibodies were diluted in PHTx: Chicken anti-βGal (1/4000, Abcam, ab9361), Rabbit anti-RBPMS (1/500, Phosphosolutions, 1830)
All secondary antibodies were diluted in PGTx or PHTx: Donkey anti-rabbit alexa-647, Donkey anti-rabbit Cy3, Donkey anti-chicken alexa488, Donkey anti-goat Cy3 (1/400; Jackson Immunoresearch). After washes in PBS, sections or retinas were coverslipped with Mowiol DabCo.

Imaging and Quantification:

Images were taken with a conventional fluorescence microscope (DM6000 Leica) using a 2.5X (suppl.Fig.2A) or 10X objective (suppl.Fig.2E,F) or with a Confocal microscope **(**TCS SP5 II, Leica) using a 10X (suppl.Fig.2C,D), 20X (suppl.Fig.1D-F, Suppl. Fig.2B) or 40X objective (suppl.Fig.1A-C).
For fluorogold retrograde labeling, quantitative analyses were done in cases where the retrograde injection was correctly positioned and filling the entire dLGN (n=2).
Images in each part (periphery, middle, center) of the 4 retinal quadrants of flattened retinas were taken. Using Cell Counter of Image J software, the proportion of recombined RGCs has been quantified in the 4 squares images taken in the 4 different quadrants of flattened retinas by counting the number of βGal positive cells among the fluorogold positive cells. This was done in contralateral and ipsilateral retinas to the injection site.
For beta-gal and RBPMS immunohistochemistry, we counted the number βGal+ and RBPMS+ cells (n=1) at P10.

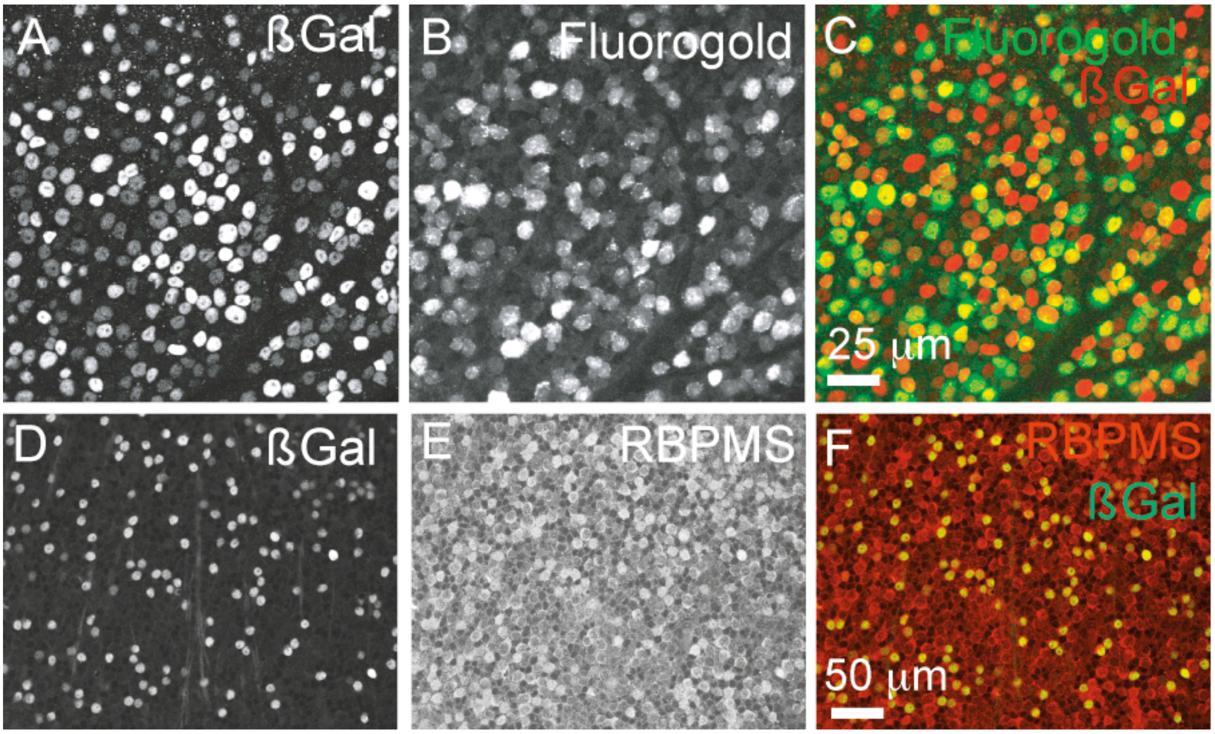

Suppl. Fig.1: βGal (A) and Fluorogold (B) co-immunostaining on a flattened whole-mount retina after retrograde labeling with Fluorogold of RGCs from the dLGN, in $Sert^{Cre/+}$ $Tau^{mGFP-NLS-LacZ/+}$ adult mice. βGal (D) and RBPMS (E) co-immunostaining on a flattened whole-mount retina in $Sert^{Cre/+}$ $Tau^{mGFP-NLS-LacZ/+}$ P10 mice. (C,F) Merged image of the two immunolabeling.

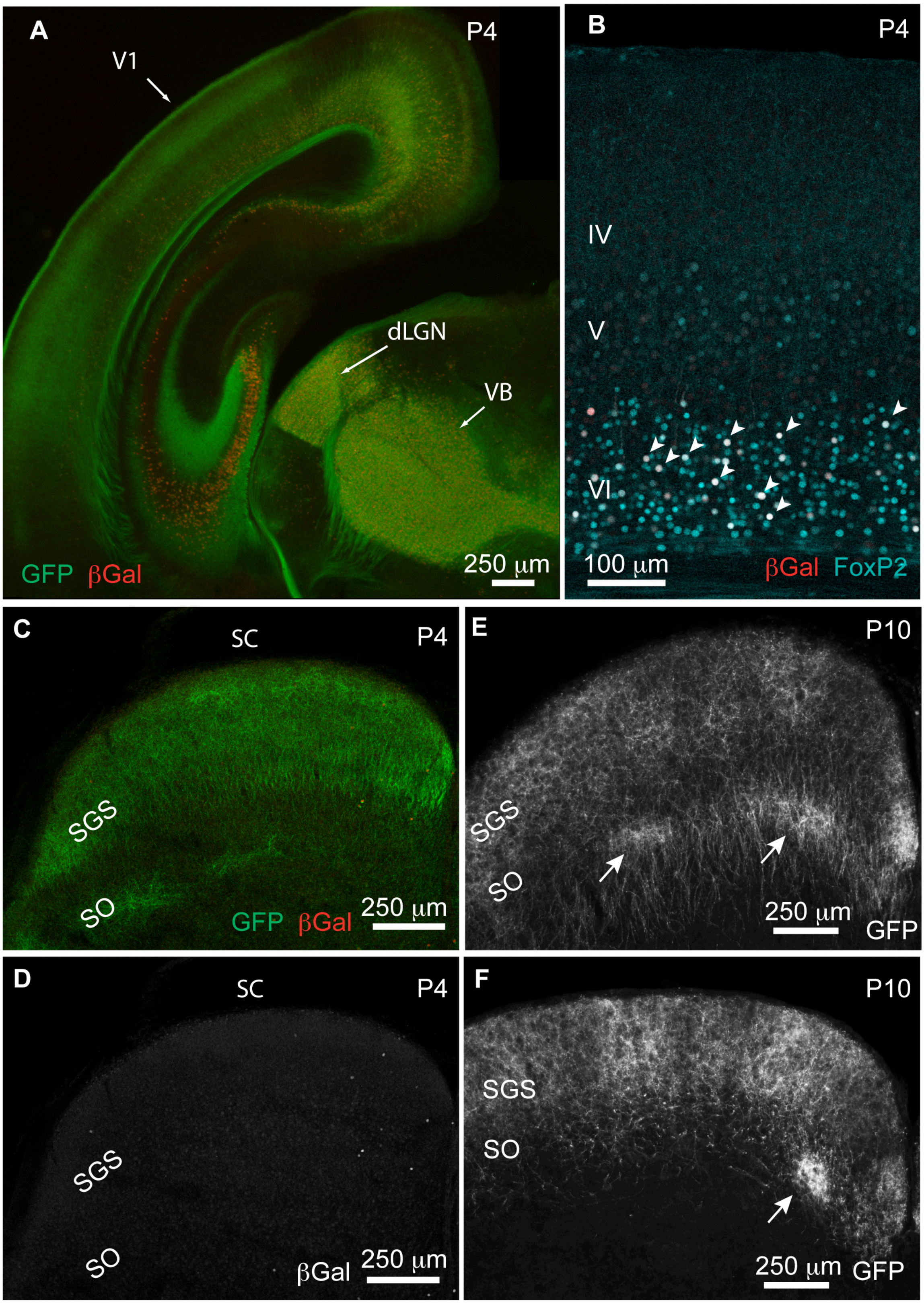

Suppl. Fig.2: (A) GFP (green) and βGal co-immunostaining in coronal sections of $Sert^{Cre/+}$ $Tau^{mGFP-NLS-LacZ/+}$ P4 mice. In the dLGN, most cells are beta-Gal+ cells at P4 and GFP labeling corresponds to incoming retinogeniculate and outgoing geniculocortical axons and

not to corticogeniculate axons as these axons have barely reached the dLGN at this age . Thalamocortical axons are clearly visible in the primary visual cortex (V1). Note that the VB (somatosensory thalamus) is also βGal+ and GFP+. (B) In the primary visual cortex, βGal (red) and FoxP2 (cyan) are co-expressed in several layer VI neurons in V1 (arrowheads). (C-F) As layer VI neurons send their axon to the thalamus, some corticogeniculate axons are probably expressing Cre in our $Sert^{Cre/+}$ mice, however they have barely reached the dLGN by P4. Although beta-Gal+ staining is absent in the SC (C,D), many GFP fibers (green or white) are seen that correspond to the retinocollicular axons innervating the superior colliculus (SC) in both the SGS and SO layers corresponding respectively to contralateral and ipsilateral laminas at P4 (C,D) and P10 (E,F) and in rostral (E) or caudal sections (F) of $Sert^{Cre/+}$ $Tau^{mGFP\text{-}NLS\text{-}LacZ/+}$ mice. This confirms our observation in the retina that RGCs from the entire retina are recombined and not only ispilateral RGCs.
